# An Optogenetic Demonstration of Motor Modularity in the Mammalian Spinal Cord

**DOI:** 10.1038/srep35185

**Published:** 2016-10-13

**Authors:** Vittorio Caggiano, Vincent C. K. Cheung, Emilio Bizzi

**Affiliations:** 1 McGovern Institute for Brain Research and Department of Brain and Cognitive Sciences, Massachusetts Institute of Technology, Cambridge, MA, 02139, USA; 2 School of Biomedical Sciences, The Chinese University of Hong Kong, Hong Kong SAR, China

## Abstract

Motor modules are neural entities hypothesized to be building blocks of movement construction. How motor modules are underpinned by neural circuits has remained obscured. As a first step towards dissecting these circuits, we optogenetically evoked motor outputs from the lumbosacral spinal cord of two strains of transgenic mice – the Chat, with channelrhodopsin (ChR2) expressed in motoneurons, and the Thy1, expressed in putatively excitatory neurons. Motor output was represented as a spatial field of isometric ankle force. We found that Thy1 force fields were more complex and diverse in structure than Chat fields: the Thy1 fields comprised mostly non-parallel vectors while the Chat fields, mostly parallel vectors. In both, most fields elicited by co-stimulation of two laser beams were well explained by linear combination of the separately-evoked fields. We interpreted the Thy1 force fields as representations of spinal motor modules. Our comparison of the Chat and Thy1 fields allowed us to conclude, with reasonable certainty, that the structure of neuromotor modules originates from excitatory spinal interneurons. Our results not only demonstrate, for the first time using optogenetics, how the spinal modules follow linearity in their combinations, but also provide a reference against which future optogenetic studies of modularity can be compared.

Movement generation for accomplishing even simple, routine task goals involves complex calculations that the central nervous system (CNS) must perform in order to coordinate the activations of the hundreds of muscles. As such, neural circuitries in the motor system are believed to be structured in a way that implements a certain simplifying principle, one that enables motor commands to be specified accurately and quickly in spite of the computational complexities inherent in the nervous and musculoskeletal systems. Understanding how this simplifying principle is represented and executed by the CNS has remained a motivating question in motor neuroscience. According to one proposal, this simplifying principle involves generating motor commands by flexibly combining discrete neuromotor modules underpinned by interneurons in the spinal grey matter (reviewed in refs 1, 2, 3, 4). It has been argued that spinal interneurons are organized into modules, each of which activates a set of muscles together as a “muscle synergy”, and that these modules can be linearly combined in different proportions so that a vast variety of functional muscle patterns can be generated with just a few modules. The possibility of combination in this model of motor modularity presumably makes motor planning, control, and learning simpler and easier^5^, at least for the commonly executed tasks in the movement repertoire.

How exactly the neuromotor modules are represented in the spinal cord and how the different types of spinal neurons are assembled to permit the expression and combination of modules have remained obscure to date. In previous experiments conducted in the spinalized bullfrog, electrical microstimulation was used to elicit modules that were represented as spatial fields of isometric force recorded at the ankle; these force-field modules could be summed linearly to produce a new force field through co-stimulation of the spinal loci that gave rise to the separately-evoked fields^6^,^7^,^8^,^9^. Saltiel *et al*.^10^,^11^ later found that modules represented as muscle synergies could be elicited using focal *N*-methyl-D-aspartate (NMDA) iontophoresis, and importantly, these same muscle synergies could be sequentially conjugated to reproduce locomotor muscle patterns^12^. The above studies, together with others based on spinal spike recordings (e.g. ref. 13), have provided good evidence that neuromotor modules are shaped by spinal interneurons. But the stimulation techniques employed in these pioneering works have not allowed a more detailed dissection of the components of the circuitry that underlies the activation and combination of the neuromotor modules.

The advent of optogenetics (reviewed in refs 14 and 15) offers the prospect of circuitry dissection through selective stimulation of particular neuronal types. In a transgenic mouse, the expression of the channelrhodopsin-2 (ChR2) transgene can be controlled under the promoter of any gene of interest, making it possible to use light to selectively activate only the neurons expressing that particular gene. By comparing the light-evoked motor outputs of multiple strains of mouse with ChR2 expressed in different neurons, it may be possible to deduce how these different neurons are wired together in the spinal circuitry that controls movement. This knowledge not only provides the neurophysiological basis to any putative motor control principles (such as the notion of motor modularity), but should also inform how these principles should be refined in their formulations for a more nuanced understanding, and for more predictive power.

In this study, as a first step towards unveiling the spinal circuitry that gives rise to neuromotor modules and their computational properties, we explored the use of optogenetics to elicit motor modules from the spinal grey matter. We applied optical stimulations to the lumbosacral spinal cord of two strains of transgenic mouse, with ChR2 selectively expressed in cholinergic neurons and putatively excitatory neurons, respectively. Our comparison of the motor outputs from these two strains allowed us to conclude, with reasonable certainty, that the structure of neuromotor modules originates from excitatory spinal interneurons. Our results here should also provide a reference point against which future optogenetic studies of motor modularity can be compared.

## Results

### Optogenetic stimulation of the spinal cord of the Chat and Thy1 mice produced different force patterns

**Figure 1:**
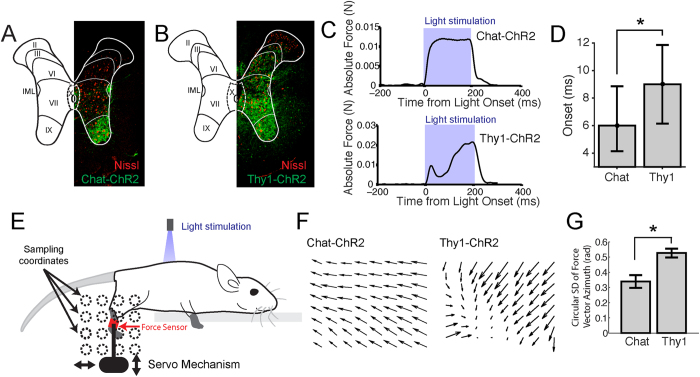
Experimental setup and design. (**A**) Immunostaining of Chat spinal slices indicates that the range of ChR2 expression (green) included neurons in the ventral horn (laminae VIII and IX) and the intermediolateral nucleus (IML), neurons that were presumably cholinergic. (**B**) Immunostaining of Thy1 slices indicates ChR2 expression through most of the spinal grey matter, with expression being the strongest in the intermediate zone (lamina VII) where interneurons monosynaptically connected to the motoneurons of different muscles are most dense. (**C)** Example time traces of ankle force optogenetically elicited from the lumbosacral spinal cord of Chat (top panel) and Thy1 (bottom panel) mice. The traces of absolute force magnitude (defined over the X-Y plane) shown are averages across data evoked from multiple ankle locations in the workspace (N = 15 for Chat; N = 20 for Thy1), but from stimulations applied to the same spinal locus. Blue shadings indicate the time windows in which optical stimulations were applied. (**D**) The Chat mouse exhibited a shorter delay of force emergence after optical stimulation onset as compared with the Thy1 mouse (*U-test, p < 0.01; mean ± SD). (**E)** The motor output for any spinal stimulation locus was represented as a spatial force field. Isometric hind-limb forces optogenetically evoked at any spinal locus were recorded as the ankle was moved to different workspace locations by an automatic servo mechanism. (**F**) Examples of spatial force fields recorded from the setup depicted in *E*, derived from the Chat (left panel) and Thy1 (right panel) mouse strains. (**G**) In general, the Chat force fields tended to be composed of parallel vectors, while the Thy1 force fields, non-parallel vectors. This difference in structure is supported by the result that the variability of force-vector direction within each field, quantified by the circular standard deviation of the vectors’ azimuth angles, was much smaller in the Chat-fields than in the Thy1-fields (*, U-test, p < 0.01; mean ± SE).

One major goal of this study is to understand how excitatory spinal interneurons contribute to the generation of force outputs through their selective stimulations. We are also interested in stimulating the motoneuronal pools along the spinal cord, so that the resulted force fields could become a comparison set for the force fields obtained from interneuronal stimulation. Activation of motoneurons was achieved by optical stimulation of the spinal cord of a transgenic mouse in which ChR2 was selectively expressed in cholinergic neurons (strain Chat-COP4*H134R/EYFP,Slc18a3; hereafter referred to as Chat) (Fig. 1A). To target excitatory interneurons, we used a transgenic mouse in which ChR2 was expressed via the Thy1 promoter (Thy1-COP4/EYFP, hereafter referred to as Thy1). In the Thy1 mouse, ChR2 has been previously shown to be expressed in excitatory neurons (e.g., layer-5 projection neurons in the motor cortex)^16^. In the spinal cord, we found ChR2 to be expressed most strongly in neurons in the intermediate zone, though expression in the ventral horn was also observed (Fig. 1B). Although excitatory glutaminergic neurons are present across all laminae of the spinal cord^17^, interneurons monosynaptically connected to the motoneurons of different muscles are more dense at the intermediate zone of the spinal cord (see ref. 18). We believed pre-motor interneurons are the neurons most involved in the observed effects.

We first used a previously developed *in vivo* preparation^19^ to test if non-invasive optical stimulations of the spinal lumbosacral segments were able to trigger motor responses. In this preparation, we monitored the resulting hind-limb isometric force by means of a force sensor attached to the ipsilateral ankle. In both Chat and Thy1 lines, light stimulation of the lumbosacral spinal cord evoked hind-limb forces (Fig. 1C). Nevertheless, forces were evoked with different delays in the two strains. In the Chat mouse, when the ankle was fixed at resting position and when the minimum necessary laser power was used, force emerged on average at ~6 ms from light onset; in the Thy1 strain, force was detected at ~9 ms from light onset (U-test, p < 0.01; Fig. 1D). The fact that we were able to evoke forces in the Thy1 mouse with delays longer than those observed in the Chat mouse suggests the following points. First, optical stimulations applied to Thy1 do not just involve direct activations of motoneurons because its force onset time was later than that in Chat when only motoneurons were directly activated. Second, in the Thy1 mouse, the force produced likely owes its origin to activation of excitatory interneurons. Indeed, selective activation of inhibitory spinal interneurons does not produce any motor output^19^. Also, Thy1 forces were evoked at weaker power intensities (about half) than the ones required for evoking forces in Chat mice, indicating, for Thy1, an activation of neurons in layers more superficial than the ventral horn where the motoneurons are located. Thus, these two transgenic mouse strains appear to be complementary with respect to the population of neurons involved in the production of motor outputs.

**Figure 2:**
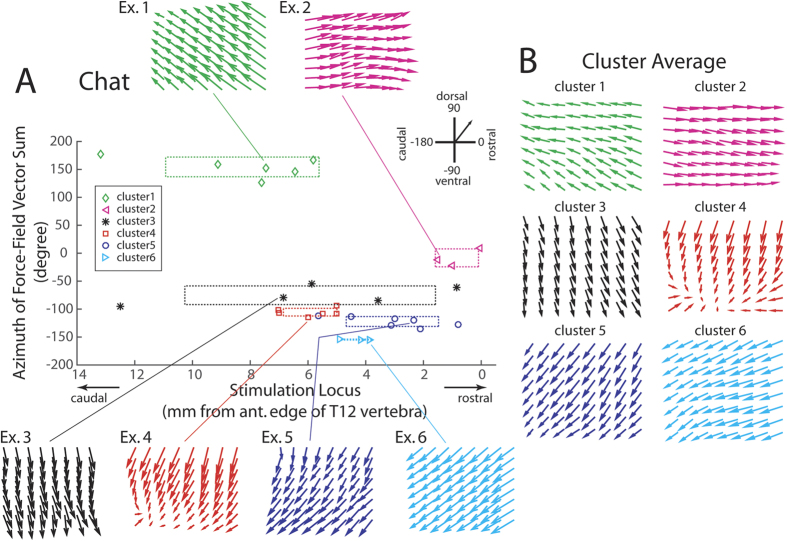
Topographic organization of spinal force fields in the Chat mouse. (**A**) We summarized force fields obtained from 5 mice, elicited using a single laser at the lowest power used at each locus (N = 30). Each force field is represented in the figure by the azimuth angle of the vector sum of all vectors in the field (y-axis), and by the spinal stimulation locus located with respect to the anterior edge of the T12 vertebra (x-axis). The Chat force fields were grouped into 6 clusters by our procedure based on *k*-means and the silhouette values. Individual force-field examples for all clusters are shown (Ex. 1 to Ex. 6). For each cluster, dotted lines mark the cluster mean ± SD along both the horizontal and vertical directions. (**B)** The average force fields for the 6 clusters shown in (**A**).

**Figure 3:**
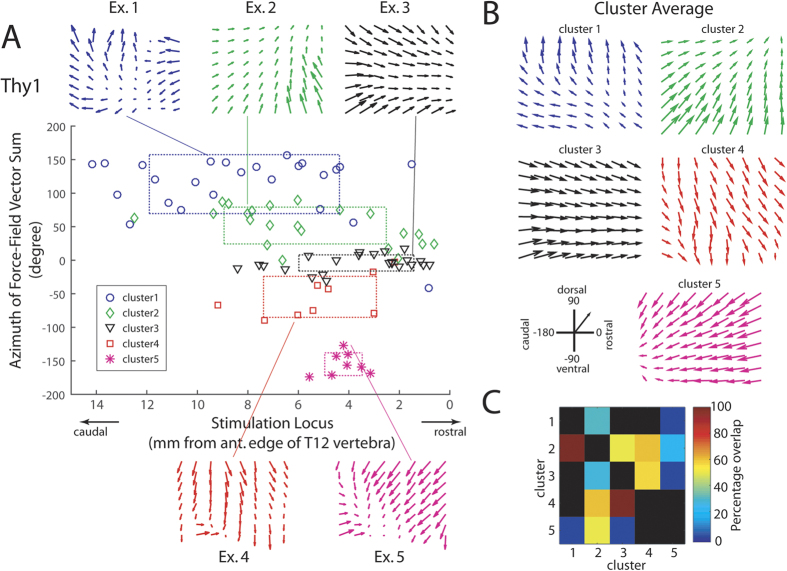
Topographic organization of spinal force fields in the Thy1 mouse. (**A**) We summarized force fields obtained from 10 mice, elicited using a single laser at the lowest power used at each locus (N = 87). Each force field is represented in the figure by the azimuth angle of the vector sum of all vectors in the field (y-axis), and by the spinal stimulation locus located with respect to the anterior edge of the T12 vertebra (x-axis). The Thy1 force fields were grouped into 5 clusters by our procedure based on *k*-means and the silhouette values. Individual force-field examples for all clusters are shown (Ex. 1 to Ex. 5). For each cluster, dotted lines mark the cluster mean ± SD along both the horizontal and vertical directions. (**B**) The average force fields for the 5 clusters shown in (**A**). (**C**) The percentage overlap between the spatial ranges of stimulation loci of every pair of clusters was calculated for each animal, and averaged across animals. These percentage values are shown here as a heat map (blue = 0 overlap; red = 100% overlap). For example, the colour shown in the box at row 2, column 1 denotes the percentage of the range of cluster 2 that overlapped with the range of cluster 1. Black means there was insufficient or no data for the cluster pair for this evaluation.

Next, in order to understand the organization and contribution of the excitatory interneurons versus the motoneurons to motor output, we analysed the forces field resulting from stimulating the same locus in the lumbosacral spinal cord as the ankle was fixed at different positions in the workspace via a servo mechanism (Fig. 1E; see Methods). We observed that in the two strains, the resulting force fields were qualitatively different. In the Chat mouse, force fields tended to be composed of parallel vectors (Fig. 1F, left panel; Fig. 2) while in the Thy1 mouse, force fields tended to be composed of non-parallel vectors (Fig. 1F, right panel) that were usually convergent (Fig. 3A, Ex. 2 to 5), but sometimes divergent (Fig. 3A, Ex. 1). This difference in structure between the Chat- and Thy1-fields is further supported by the result that the variability of force-vector direction within each field, quantified by the circular standard deviation of the vectors’ azimuth angles, was much smaller in the Chat-fields than in the Thy1-fields (U-test, p < 0.01) (Fig. 1G; Fig. S2B). Consistent with this statistical result, we found that when the variance of the azimuth angles of each Chat field (N = 30) was compared with that of each Thy1 field (N = 87) using the Ansari-Bradley test of dispersion, among the 30 × 87 = 2610 comparisons, 2085 of them (80%) achieved a p-value < 0.05; and 1845 (71%), a p-value < 0.01. In Chat, 57% of the stimulation loci produced parallel force fields, but in Thy1, only 33%. The force fields from the Thy1 animals are in this sense more complex in structure than those from the Chat animals.

### Optogenetic spinal stimulation of the Chat mouse resulted in force fields with parallel vectors

We next proceeded to investigate how in the two strains different force fields are topographically organized in the lumbosacral spinal cord. We summarized how the Chat-force fields (elicited using a single laser at the lowest power at each locus; N = 30 from 5 mice) varied with the stimulation locus in Fig. 2A, in which each force field obtained is represented, on the y-axis, by the azimuth angle of the vector sum of all vectors in that field. The Chat-force fields could be grouped into 6 clusters, all of which were mostly or wholly composed of parallel force vectors (Fig. 2B). Fields with anteriorly directed vectors were elicited at rostral spinal loci (cluster 2; Fig. 2A, magenta) whereas fields with posteriorly directed vectors, at more caudal loci (cluster 1; Fig. 2A, green); the stimulation loci in between these two clusters produced force fields directed either ventrally (clusters 3 and 4) or postero-ventrally (clusters 5 and 6). Interestingly, we did not observe any dorsally directed force fields. Our results from the Chat mouse are consistent with the earlier simulation results based on the bullfrog that with the particular topography of the motoneuronal pools in the spinal cord, activating a small neighbourhood of motoneurons (often containing motoneurons innervating several muscles) likely produces force fields with parallel vectors^7^.

### Optogenetic spinal stimulation of the Thy1 mouse resulted in force fields with non-parallel vectors

We next attempted to reveal the topographic organization of the force fields along the lumbosacral spinal cord by eliciting hind-limb forces with optogenetic spinal stimulation of the Thy1 mouse. The Thy1-fields derived from a single laser at the lowest power at each locus (N = 87 from 10 mice) could be categorized into 5 distinct clusters (Fig. 3B). Represented the most rostrally on the spinal cord was a cluster with anteriorly directed forces (cluster 3; Fig. 3A, black). This was followed, in the caudal direction, by a cluster with antero-dorsally directed forces (cluster 2; Fig. 3A, green), and then, two clusters with downward forces (cluster 4; Fig. 3A, red) and postero-ventrally directed forces (cluster 5; Fig. 3A, magenta), respectively. The most caudally represented cluster comprised divergent vectors that were oriented postero-dorsally (cluster 1; Fig. 3A, blue). Note also that unlike the Chat mice in which force field with dorsally oriented vectors was not observed (Fig. 2A), the Thy1 mice produced force fields with azimuth angles that spanned the entire anteroposterior and dorsoventral ranges (Fig. 3A). In this sense, the set of Thy1 force fields are more diverse in structure than the set of Chat force fields.

To characterize the topographic representations of the force-field clusters in more detail, we calculated, for *each* Thy1 mouse and for *each* cluster, the percentage overlap between its spatial range of stimulation loci and that for every other cluster (Fig. 3C). While some cluster pairs such as clusters 1 and 5 and 3 and 5 were totally non-overlapping, other pairs such as clusters 1 and 2 and 3 and 4 exhibited a high degree of overlap in their representations. Thus, the overlaps between the ranges for the force-field clusters evident in Fig. 3A were not just merely due to inter-animal variability in the spinal topographic map.

### The Thy1-equilibrium points had a larger spatial distribution than the Chat-equilibrium points

When examining the force fields elicited by a single laser beam, we found that some force fields from either mouse types were characterized by the presence of a single equilibrium point (EP) at which no net active force could be elicited at the ankle by optogenetic stimulation. In some other force fields, the vectors along one of the four borders of the workspace were sufficiently convergent that they defined a “virtual” EP just outside the workspace, near the workspace boundary, at the point where the vector lines intersected^20^. Among the Chat-force fields derived from a single laser beam (N = 93), EP was identified in 11.8% (N = 11), and virtual EP, in 8.6% (N = 8). Among the Thy1-force fields derived from a single laser beam (N = 212), EP was identified in 16.5% (N = 35), and virtual EP in 17.0% (N = 36). Thus, there was a higher percentage of fields containing EPs or virtual EPs among the Thy1-fields (33.5%) than among the Chat-fields (20.4%).

**Figure 4:**
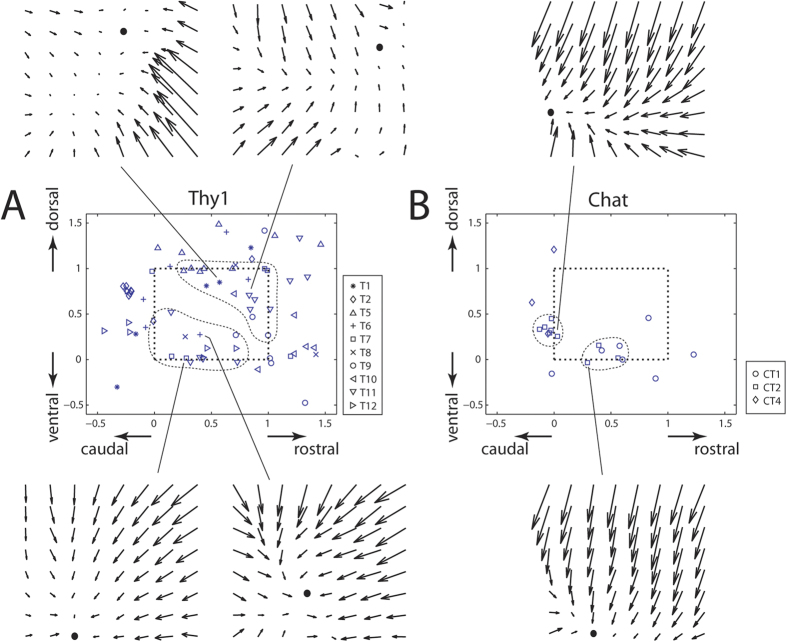
Equilibrium points (EP) in force fields evoked from the Chat and Thy1 animals. (**A**) Among all the Thy1 force fields we obtained using a single laser (N = 212 from 10 mice), EPs and “virtual” EPs located just outside the workspace were detected in 71 of them (33.5%). This figure summarizes the spatial distribution of the Thy1 EPs and virtual EPs, with the EPs from each mouse (whose name is prefixed by the letter “T”) denoted by a unique symbol. The workspace of the hind-limb is demarcated by dotted lines. It is visually apparent that there were 2 spatial clusters of EPs within the workspace, one located antero-dorsally, and another, postero-ventrally. Four specific examples of force fields with EPs are also shown. In each, the EP is marked with a black dot. (**B**) Among all the Chat force fields we obtained using a single laser (N = 93 from 5 mice, with 2 mice producing no force field with EP), EPs and virtual EPs located just outside the workspace were detected in 19 of them (20.4%). The EPs and virtual EPs from each mouse (whose name is prefixed by the letters “CT”) are denoted by a unique symbol. All Chat EPs were spatially circumscribed within 2 small regions near the workspace boundary, and for each, a specific example of force field with EP is shown. In both panels (A,B) since many EPs detected were located at the same nodes within our 8 × 8 force-vector grid, to visually clarify the spatial density of the EPs, we have added a very small Gaussian noise to both the x- and y-coordinates of the EPs when producing this figure.

The EPs from the Thy1 mice occupied a much wider area within the hind-limb workspace than the Chat-EPs (Fig. 4). In fact, almost all Chat EPs were spatially circumscribed within 2 small regions near the workspace boundary, one located ventrally, and another, postero-ventrally (Fig. 4A). On the other hand, the Thy1-EPs distributed themselves across more than half of the workspace area (Fig. 4B). It was visually apparent that there were 2 spatial clusters of Thy1-EPs, one located antero-dorsally, and another, postero-ventrally (Fig. 4B). These EP groups correspond roughly to final limb-endpoint positions of trajectories for limb flexion and limb extension, respectively.

### Optogenetically evoked fields in both Thy1 and Chat mice displayed linear combination

**Figure 5:**
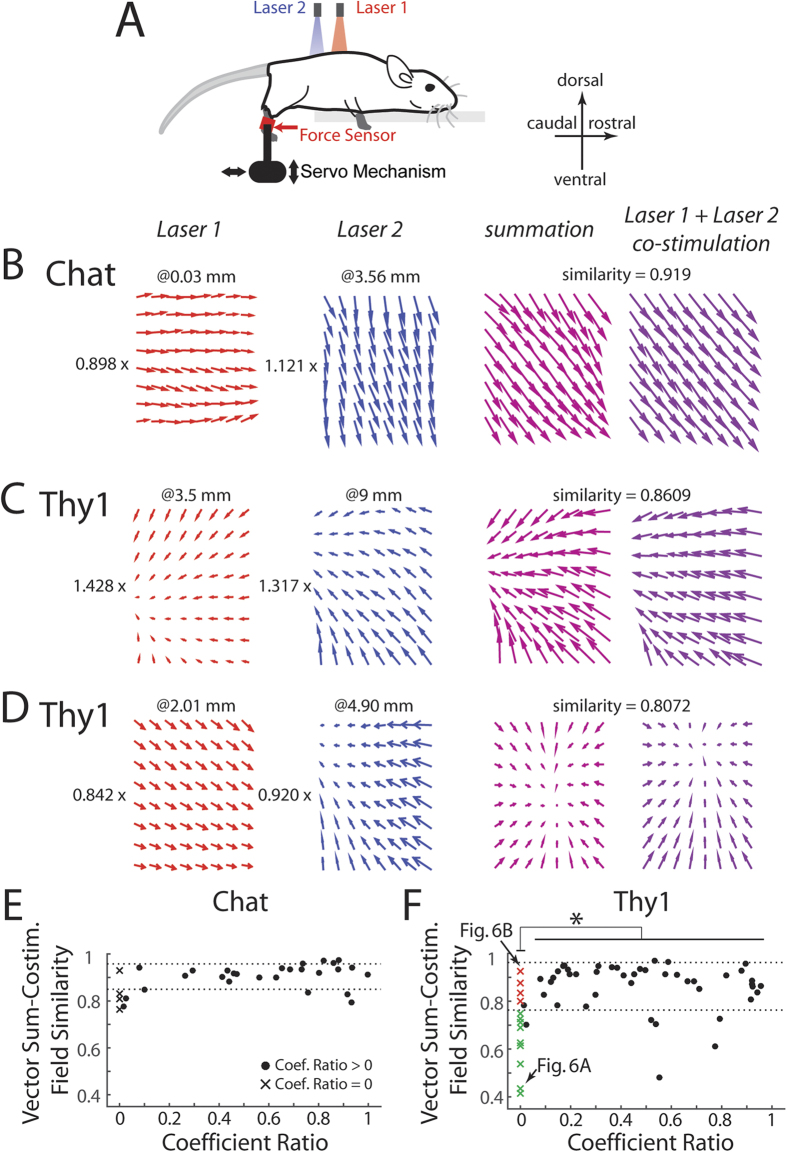
Optogenetically evoked fields in both Thy1 and Chat displayed linear combination. (**A**) To assess whether the property of linear combination holds for force fields optogenetically derived, we elicited force fields with co-stimulation from two laser beams, placed at separate spinal loci. (**B**) An example from a Chat mouse showing how the force field produced by co-stimulation (rightmost panel) can be reconstructed by linearly combining the force fields separately evoked at the individual loci (laser 1 and laser 2). The coefficients for this linear combination, shown to the left of the individual fields, were found by linear regression. The locations of the spinal stimulation loci, measured in mm with respect to the anterior edge of the T12 vertebra, are shown on top of the individual force fields. Similarity between the reconstructed and co-stimulation force fields was quantified using equation (5) stated in Methods. (**C,D**) Two examples from two different Thy1 mice demonstrating how spinal force fields could be linearly combined to explain fields derived from co-stimulation. (**E,F**) Similarity between the reconstructed and co-stimulation force fields was plotted against the coefficient ratio, defined as the smaller of the two combination coefficients divided by the larger coefficient, for the Chat (panel E) and Thy1 (panel F) mice, respectively. A coefficient ratio of 0 (x) implies that a “winner-take-all” model produces a better fit. Importantly, considering the cases with coefficient ratio > 0 (•), the average similarity values for the Chat and Thy1 were not significantly different from each other (p > 0.05). Also, in both mouse strains, when the coefficient ratio was > 0, similarity was not dependent on the coefficient ratio (p > 0.05), further suggesting the generality of the linear-combination model of force-field modules. In Thy1 (panel F), when coefficient ratio = 0, some co-stimulation fields were well explained by the winner (red x), but others were explained poorly (green x). Horizontal dotted lines mark mean ± SD of the similarity values for cases with coefficient ratio > 0. In (**F**) two specific data points with a coefficient ratio of 0 (marked with arrows) are shown in Fig. 6 as examples of winner-take-all.

A defining hallmark of the current theory of motor modularity is that motor outputs produced by distinct modules can be linearly summed in a flexible manner to produce diverse new motor patterns. In previous studies of spinal force fields, this property of linear summation was demonstrated by a comparison of the field elicited by co-stimulation of two spinal loci, and the field computed by summing the two fields separately evoked at the same loci^6^. Here, we adopt a similar approach to assess whether fields optogenetically evoked by two laser beams also display linear summation in both mouse types (Fig. 5A).

Previous studies have shown that linear summation of fields derived from direct stimulation of individual muscles is a very robust property in multiple vertebral musculoskeletal systems^8^. Even though it is likely that each laser stimulation applied to the Chat mouse in this study activated motoneurons from the motoneuronal pools of multiple muscles, as suggested by the very fact that the Chat fields were mostly composed of parallel vectors (Fig. 2) (see Fig. 14 of ref. 7), the robustness of linearity in single-muscle fields led us to anticipate that linear vector-field summation should also be seen in the Chat mouse. We found, with our regression analysis, that in 27 of 31 of the Chat cases (87.1%), both single-laser fields were assigned non-zero scaling coefficients (i.e., coefficient ratio >0; ratio defined as the smaller coefficient divided by the larger coefficient) when they were summed to best-explain the co-stimulation field. Within the set of cases with non-zero coefficient ratio, we observed an extremely good match between the summed and co-stimulation fields (Fig. 5B). In fact, the similarity between these two fields achieved an average of 0.90 ± 0.05 (mean ± SD), with a similarity value of 1 corresponding to identical fields (Fig. 5E). This result obtained from the Chat may then be regarded as a baseline similarity level against which the Thy1 result may be compared.

As for Thy1, our regression analysis found a smaller percentage of cases with non-zero scaling coefficients for both single-laser fields, as compared with Chat (45 of 58; 77.6%). However, the correspondence between the computed field obtained by summing the single-laser fields and the co-stimulation field was as good as that observed in Chat (Fig. 5C,D). For the Thy1 cases with a non-zero coefficient ratio, the average similarity between the summed and co-stimulation force fields was 0.86 ± 0.10 (mean ± SD), a value smaller than the Chat value, but not significantly different from it statistically (U-test, p > 0.05) (Fig. 5F). We concluded that linear combination holds for optogenetically evoked force fields in both Chat and Thy1.

We note here that when the coefficient ratio was non-zero, the similarity level did not vary as a function of the coefficient ratio in both Chat (Fig. 5E, •) (Pearson’s r, p > 0.05) and Thy1 (Fig. 5F, •) (p > 0.05) mice. Also, for both mouse types, the coefficient ratio did not depend on the difference in the laser power used in the two stimulation sites (Kruskal-Wallis, p > 0.05), nor on the distance between the two spinal stimulation loci (p > 0.05).

### Some co-stimulation fields from the Thy1 mouse were better explained by a winner-take-all model

We showed, in the above section, that linear summation is a viable model that explains how spinal force fields elicited by optogenetics are combined to produce a new field. An alternative possibility is that the field evoked by co-stimulation is identical to one of the two fields evoked by a single laser beam^8^. Such a scheme would constitute a “winner-take-all” model: The single-laser field that is also expressed as a co-stimulation field is the “winner” that prohibits any output of the other unexpressed field. In our analysis, the winner-take-all possibility is implicated when our linear regression procedure finds that to best explain the co-stimulation field, the scaling coefficient for one of the single-laser fields would have to be 0 (i.e., coefficient ratio = 0). In other words, the single-laser field with the non-zero coefficient should be the “winner”.

Among the Chat cases we analysed, 4 of 31 (12.9%) were better explained by the winner-take-all model. For these few cases in Chat, the similarity between the winner and co-stimulation fields was as good as that observed in the linear-summation cases (Fig. 5E, x) (U-test, p > 0.05).

**Figure 6:**
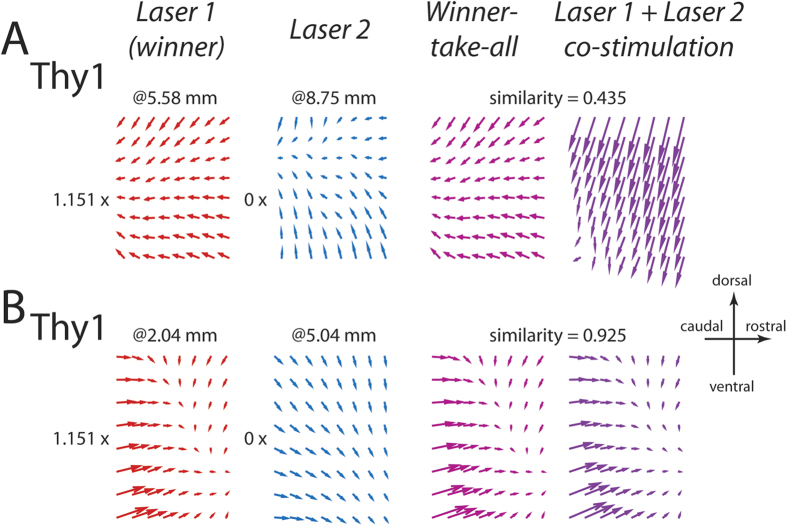
Examples of Thy1 co-stimulation fields better explained by a winner-take-all model. (**A**) In this example, our regression analysis found that a coefficient ratio of 0 was the best fit; but the “winner” field and the co-stimulation field matched poorly (similarity of 0.435). (**B**) An example in which the “winner” field and the co-stimulation field matched extremely well (similarity of 0.925).

In Thy1, 13 of 58 cases (22.4%) appeared to be better fitted by the winner-take-all model. Interestingly, this percentage agrees very well with the frequency of winner-take-all cases previously estimated by Mussa-Ivaldi *et al*.^8^ using electrical micro-stimulation applied to the frog spinal cord (19.5%). For these 13 cases, the similarity between the winner and the co-stimulation fields (0.69 ± 0.16) was, on average, lower than the similarity achieved when coefficient ratio was greater than 0 (Kruskal-Wallis, p < 0.01; Fig. 5F*). In fact, among these Thy1 cases showing a zero coefficient ratio, the co-stimulation field was well explained by the winner in 4 of 13 cases (30.8%) (Fig. 5F, red x), with their similarity values lying within the range of values from cases with coefficient ratio >0 (range defined by mean ± SD). For the remaining 9 cases (69.2%), neither the “winner” nor any linear combination of the separate fields could explain the co-stimulation field well (Fig. 5F, green x). As examples, we show in Fig. 6 one Thy1 case in which the winner-take-all model explained the co-stimulation field poorly (Fig. 6A; similarity of 0.435), and another case in which the winner provided an extremely good fit to the co-stimulation field (Fig. 6B; similarity of 0.925).

## Discussion

In this study, we seek to elicit neuromotor modules, represented as time-invariant fields of isometric force generated at the ankle, from the murine spinal cord using optogenetics. To distinguish between the contributions of motoneurons and spinal interneurons to the structuring of the force fields, we compared fields obtained from two mouse types with ChR2 selectively expressed in cholinergic neurons (Chat) and putatively excitatory neurons (Thy1), respectively. We found that the Thy1 force fields were more complex and diverse in structure than the Chat force fields, in that the Thy1 fields comprised mostly non-parallel vectors while the Chat fields comprised mostly parallel vectors (Figs 2 and 3); that the set of Thy1 fields spanned a wider range of force directions along both the anteroposterior and dorsoventral axes than the set of Chat fields (Figs 2 and 3); and that the EPs and virtual EPs from the Thy1 fields had a wider spatial distribution than those from the Chat fields (Fig. 4). More notably, in most trials of co-stimulation carried out with two laser beams, linear combination of the separately-evoked fields explained the co-stimulation field very well in both mouse types (Fig. 5) even though in ~7% of the Thy1 cases, the winner-take-all model produced a better fit (Fig. 6). Overall, we have demonstrated, for the first time specifically using optogenetics, a topographic representation of distinct neuromotor modules in the murine lumbosacral spinal cord, and how the modules follow linearity in their combinations.

### The Thy1 force-field clusters as representations of neuromotor modules in the spinal cord

In the intact Thy1 mouse, we have identified 5 distinct clusters of force fields (Fig. 2A,B) whose corresponding regions along the spinal cord partially overlapped each other (Fig. 2A,C). We interpret these force-field clusters as representations of neuromotor modules in the spinal cord, with the rationale that their properties, to be listed below, agree with those of spinal modules previously derived using other techniques. First, unlike the Chat fields, the Thy1 fields were composed of non-parallel vectors (Fig. 1G) that were mostly convergent (Fig. 2A, Ex. 2–5). This result agrees extremely well with previous observations of vector convergence in force-field primitives elicited, using electrical microstimulation, from the spinalized frog^6^,^7^,^8^, deafferented frog^21^, decerebrated frog^22^, spinalized rat^23^, and decerebrated cat^24^. Such field convergence endows the Thy1 fields with more complexity in structure relative to the Chat fields, so that as neuromotor modules, the Thy1 fields have the potential to be combined to generate more varieties of force pattern for diverse motor tasks.

Second, our clustering procedure based on the *k*-means algorithm and the silhouette values identified 5 clusters of force fields from the Thy1 mouse, a relatively small number of clusters that compares well with the number of force-field primitives previously identified in the lumbar spinal cord. Bizzi *et al*.^6^, for example, found 4 types of field by applying electrical microstimulation to the lumbar grey matter. Using focal NMDA iontophoresis, Saltiel *et al*.^25^ likewise elicited 5 clusters of force orientations from the lumbar grey. In three follow-up studies using the same stimulation method, Saltiel *et al*.^10^,^11^,^12^ found 7 spinal modules (muscle synergies) that exhibited a partially overlapping representation along the rostrocaudal direction, similar to what we observed in the Thy1 topography (Fig. 3A). Whether a relatively small number of neuromotor modules are sufficient for generating the bewildering varieties of motor pattern needed for daily activities has remained controversial^26^,^27^,^28^. The small number of force-field types we identified here notwithstanding, each Thy1 cluster we reported may itself be composed of several more fundamental modules. It remains possible that there exists in the spinal cord a relatively large repository of different modules, each of which is specific to one or a few motor tasks; their use serves to facilitate the fast specification of a suitable muscle pattern for any task in the repertoire of commonly executed movement^3^,^29^.

Third, linear superposition of neuromotor modules, which we also observed here (Fig. 5), was a defining characteristic of previously identified modules that were represented as spinal force fields^6^,^8^,^24^,^30^. The percentage of cases in which a linear summation model fit better than a winner-take-all model reported by Mussa-Ivaldi *et al*.^8^ also agrees well with our result here (~80%) (Fig. 5F). It is noteworthy that in addition to spinally elicited force fields, motor modules evoked by cortical stimulations also sum linearly^20^,^31^,^32^. Linearity as a property of motor modules is further supported by the by now decently sized literature documenting how multi-channel electromyographic signals collected from different behaviors can be decomposed into muscle synergies by linear factorization algorithms (reviewed in refs 2,28, 33 and 34; among others). How the motor cortices, spinal networks, and the musculoskeletal system^35^ together give rise to linearity has remained obscure. But it does appear that linear summation is a general and robust property of neuromotor modules regardless of how they are elicited or represented.

It is important to note that in our results, ~20% of Thy1 co-stimulation cases were better explained by a winner-take-all model (i.e., coefficient ratio = 0). But within this group, the winner approximated the co-stimulation field well only in ~30% of cases (Fig. 6B); thus, 9 of 58 cases (15.5%) could neither be explained well by the simple linear combination model nor by the winner-take-all model (e.g., Fig. 6A). For these cases, the co-stimulation fields were likely the result of complex interactions between the interneurons, motoneurons, and sensory afferents. It is plausible that a nonlinear model of motor modularity such as those outlined in ref. 36 could account for these cases. Under what circumstances would the linear-combination model break down should be a fruitful future research direction.

### Vector convergence in Thy1 fields shaped by excitatory spinal interneurons

An important aspect in our experimental design is our comparison of results from two different transgenic mice, Chat and Thy1. In the Chat mouse, our histological examination of the spinal cord (Fig. 1A) indicates strong ChR2 expression in the ventral horn (laminae VIII and IX), the intermediolateral nucleus (lamina VII), and weak ChR2 expression in the central zone (lamina X). The cholinergic populations in laminae VIII/IX and VII obviously correspond to the motoneurons and the sympathetic preganglionic neurons, respectively. The population in lamina X are likely spinal cholinergic interneurons that function to modulate excitability of individual motoneurons, and hence do not contribute to coordination of the motoneurons for multiple muscles^37^,^38^. Thus, the force-field outputs we observed in the Chat mouse only reflect the topography of the motoneuronal pools in the lumbosacral spinal cord.

In the Thy1 mouse, our histological examination (Fig. 1B) indicates the presence of ChR2-positive neurons through most of the spinal grey matter (laminae III to X) with their expression being the strongest in the intermediate zone (lamina VII). The Thy1 protein has long been known to be expressed in the spinal grey matter^39^. Other optogenetics studies using the Thy1-ChR2 mouse have demonstrated salient ChR2 expression in excitatory neurons such as the layer-5 projection neurons in the motor cortex^40^,^41^,^42^. Some studies also found that markers of inhibitory neurotransmitters were absent in Thy1-ChR2-positive neurons^16^. Our histology and these previous demonstrations together suggest that the motor outputs we recorded in our Thy1 mice originate from activations of excitatory interneurons along with motoneurons in the ventral horn. Since the dendrites of Thy1-expressing neurons do appear to carry ChR2^16^, we think it is very likely that the connectivity between the stimulated interneurons and the motoneurons is reflected in the force field pattern, even though part of the force output no doubt result from activations of the axonal processes (see Liske *et al*.^43^).

More importantly, the selective expression of ChR2 of the Chat and Thy1 mice in two different neuronal populations allows us to strongly establish that the convergence (or divergence in a few cases) of vectors observed in the Thy1 mouse owes its origin to activations of spinal interneurons. Such differentiation of the force-output contributions of different neuronal types was not possible in earlier studies of neuromotor modules accomplished using electrical microstimulation or NMDA iontophoresis. Even though in Thy1, ChR2 expression was not confined to interneurons but was observed also in motoneurons (Fig. 1B), this lack of specificity in ChR2 expression does not prevent us from attributing vector convergence to the interneurons. Our force onset analysis (Fig. S2A) showed that at the lowest power, at which because of an exponential decay of the power intensity with the depth should not be able to activate deeper layers of the spinal cord, Thy1 forces emerged with a longer delay than the Chat forces and with more complex patterns; but as power increased, the Thy1 onset delay decreased and converged to the Chat level. This result implies that at low power, the Thy1 force fields resulted from activations of mostly interneurons that were one or a few synapses away from the motoneurons, thus the longer force delay. But at higher stimulation power, the similarity of the Thy1 and Chat onset times suggests that the laser recruited both motoneurons and interneurons in Thy1. Interestingly, we also found that the degree of vector convergence of the force fields, quantified by the circular SD of the vector azimuth angles, did not vary with the laser power in both Thy1 and Chat (Fig. S2B); also, the Thy1 SD values were higher than the Chat values (Fig. S2B). Taken together, these results argue that the activation of interneurons was sufficient to produce a force field with a level of vector convergence higher than that of the Chat fields.

The role of spinal interneurons in shaping the force fields in Thy1 is further illustrated by the following argument. Let 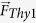 be a force field elicited from the spinal cord of the Thy1 mouse, 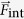
be the force-output contribution by the spinal interneurons, and 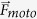
be the force-output contribution by the cholinergic motoneurons as observed in the Chat mouse. If we assume that 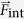 and 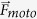 sum linearly to result in the final isometric force output^35^, we have


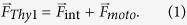


To quantify the extent of vector convergence or divergence in a field, we can compute the field’s divergence (*div*):


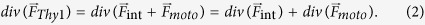


As we observed in the Chat mouse, for most of the spinal loci activation of a small neighbourhood of motoneurons produces a field of more parallel vectors (Fig. 2). Thus, for these loci, 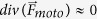. Thus,


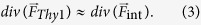


From the above derivations, we conclude that at least for the spinal loci where Chat stimulations gave rise to reasonably parallel fields, any non-zero divergence in the Thy1 field was mostly produced by activations of the spinal interneurons. Since some Chat fields were not completely parallel (e.g., cluster 4 in Fig. 2), motoneuronal contributions to non-zero divergence cannot be entirely excluded. But so long as |
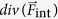
| ≫ |
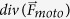
|, the above argument should still apply. Note that in equation (3), 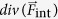 can either be zero or non-zero; thus, this argument does not *per se* contradict our observation that the Thy1 force fields, while consisting of convergent or divergent vectors most of the time, were occasionally parallel (e.g., Fig. 5D, left-most force field).

We think the spinal interneurons responsible for shaping vector convergence (or divergence) in the Thy1 mouse likely include the “motor synergy encoder” (MSE) interneurons recently identified by Levine *et al*.^18^. This is a specific, molecularly defined class of interneurons that receives inputs from the motor cortex and the sensory pathways, and at the same time drives motoneuronal activations of multiple muscles. Such a circuitry for the MSE agrees well with our earlier conclusion that the interneurons structuring the motor modules must be driven by descending motor commands from the cortex or the brain stem^44^, and modulated by sensory afferents^45^,^46^. Given that selective activation of glutaminergic neurons in the murine spinal cord is sufficient to initiate and maintain locomotor rhythm^17^, we speculate that the interneuronal networks shaping the neuromotor modules comprise MSE’s that are excitatory glutaminergic neurons, and modulated by neurons expressing other neurotransmitters including glycine and ϒ-aminobutyric acid (GABA)^18^,^19^.

### Optogenetics as a viable tool for dissecting neural circuits underlying neuromotor modules

Overall, our results obtained here with the mouse and optogenetics agree extremely well with those described in our earlier studies performed using the bullfrog/rat and electrical microstimulation. This agreement validates optogenetics as a valid stimulation technique suitable for unveiling the neural underpinnings of neuromotor modules. Indeed, in the motor control field, optogenetics has been increasingly used for understanding motor circuitries through stimulations of either the spinal cord^17^,^18^,^19^,^47^,^48^, the brain stem^49^, or the motor cortex^42^,^50^. In addition to temporal and spatial specificity of stimulation, using optogenetics in transgenic mice offers the advantage of activating only a specific class of neurons defined by the expression of any gene of interest (e.g. refs 40 and 51). A comparison of results derived from different transgenic mice should allow a dissection of how different neuronal types contribute to a motor output. For instance, in this paper, by comparing the Thy1 fields with the Chat fields, we can conclude, with reasonable certainty, that convergence or divergence of vectors in the Thy1 fields owes its provenance to activations of excitatory spinal interneurons.

In summary, in this paper we have explored the use of optogenetic stimulation for deriving a basic physiological understanding of neuromotor modules. Our comparison of results from two strains of transgenic mice led us to assert that the structure of spinal modules is likely shaped by excitatory interneurons. It is expected that the optogenetic approach presented here, when combined with the use of novel optical probes^52^, spike recordings^13^,^20^, and new statistical algorithms for extracting modules from high-dimensional data^36^,^53^, should soon allow a much more solid understanding of how neuromotor modules should be represented, and what their underlying circuitries are in the mammalian motor system.

## Materials and Methods

### Animals, surgery, and histology

Two transgenic mouse lines, Chat-ChR2-EYFP line 6 (Chat-COP4*H134R/EYFP,Slc18a3. The Jackson Laboratory stock No. 014546)^54^, and Thy1-ChR2-YFP (Thy1-COP4/EYFP; The Jackson Laboratory stock No. 007612)^40^, were used in this study. Animal care was in accordance with guidelines from the US National Institutes of Health, and approved by the Committee of Animal Care of MIT (protocol number 0913–075–16). All surgical procedures were performed under either isoflurane or ketamine/xylazine anesthesia. Necessary precautions were taken to minimize animal suffering to the extent possible.

Adult mice of either sex (~20–30 g) were anesthetized with isoflurane (1.5–2%; mixed with 0.8 L/min oxygen), and then with the ketamine (100 mg/kg) and xylazine (10 mg/kg) cocktail, injected intraperitoneally. Body temperature was maintained at 37 °C with a heating blanket (Harvard Apparatus) and heating lamps. The dorsal skin surface above the thoracic and lumbosacral spine was shaved, and then cleaned with three alternating washes of ethanol (70%) and povidone-iodine. Following a midline skin incision, the subcutaneous soft tissues and back muscles were retracted to expose the lateral masses of the thoracic and lumbosacral vertebrae. We then scraped the bone clean on the top and the sides. We severed the muscle tendons attached to the vertebrae using surgical scissors, and then mounted the vertebrae onto a stereotaxic frame through custom-made spinal clamps. The vertebral laminae of T12, T13, and L1 (exposing spinal segments L2-L6 and S1), or T13, L1, and L2 (exposing spinal segments L4-L6 and S1–S4)^55^ were removed by a pair of spring scissors. After laminectomy, spinal anatomical landmarks were identified according to the pattern of anastomosis of the blood vessels. Excess bleeding was stopped by means of cotton swabs and gel foam. The exposed spinal cord was covered with either a thin layer of agarose (2% in PBS) or saline solution to keep the spinal cord moist, and to reduce the impact of heat produced by light stimulation.

After the experiment, the mouse was perfused transcardially with cold PBS followed by paraformaldehyde (PFA) (4% in PBS). The spinal cord was extracted and kept in PFA (4%) at 4 °C overnight, and then left in sucrose (30%). Slices of the spinal cord were collected in a cryostat at thickness of 20 or 40 μm. For immunostaining, each slice was placed in PBST (PBS + 0.2% Triton X-100) with serum (10%) for 2 h at room temperature, and then incubated overnight with primary antibody at 4 °C (chicken/rabbit anti-GFP 1:500, Invitrogen). Slices then underwent three wash steps, 10 min each, in PBST, followed by incubation (2 h) with secondary antibody (1:200 AlexaFlour 488 anti-chicken/rabbit, Invitrogen; Nissl stain - NeuroTrace Deep Red 1:1000, Life Technologies). Slices were then incubated and mounted with DAPI (Vectashield) on microscope slides, and imaged using a confocal microscope (Zeiss LSM 5 Pascal Exciter).

### Laser stimulation

We employed two diode-pumped solid state blue lasers (473 nm; maximum power 200 mW; Laser Century) coupled to a 200-μm core optical fiber (ThorLabs) via FC/PC connectors. Tip of the optical fiber was positioned just above the spinal cord (200–300 μm) by means of a movable device, ipsilateral to the side of force collection (~150 μm lateral to midline). Typically, light pulses of 20 to 40 mW were utilized. For each spinal stimulation locus, we first established the minimum power needed to elicit a force response, and obtained a force field using this power before we increased the power in increments of 2.5 to 15%. Onset time of stimulation, stimulation frequency (set at 100 Hz for all trials), pulse width (5 ms for all trials), and duration of the stimulation (200 ms for all trials) were controlled by custom Matlab scripts.

### Recording hind-limb isometric force from ankle

In the anesthetized mouse preparation described above, the isometric force of the hind-limb evoked by optical stimulation was measured by a six-axis force transducer (ATI sensor; 20 kHz), linked to the hind-limb of the animal using a cuff wrapped securely around the ankle. The transducer was mounted on a servo positioning device (Zaber; model T-LSM100) that allowed the ankle to be fixed in any position within the workspace of the hind-limb. We controlled and randomized the position of the ankle by means of customized scripts written in Matlab.

In each trial, the onset time of force emergence was detected as follows. The magnitude of the absolute force evoked was first computed from the x- and y-components of the force (corresponding to the anteroposterior and dorsoventral axes, respectively) as measured by the force transducer. The baseline force, defined as the average force recorded during the 20-ms window before stimulation onset, was then computed, and the maximum force magnitude attained in the trial (over all ankle locations) was identified. A force threshold was then defined as either the baseline plus 3 times the standard deviation (for the experiments where the leg was not moved in the workspace) or as the baseline force plus 3% of the difference between the maximum force and baseline force. Force onset time was defined as the first time point, after laser stimulation onset, at which the force magnitude exceeded the threshold. For both mouse strains, we analysed force onset time in two different sets of animals. In the first set (3 Chat mice; 5 Thy1 mice), we obtained a force onset estimate by stimulating with the minimum power necessary for evoking hind-limb force while the ankle was fixed at its normal resting position (Fig. 1D,E). In the second set (5 Chat mice; 10 Thy1 mice), at the same spinal stimulation locus and laser power, we recorded isometric forces with the ankle fixed at different locations in the workspace to derive a spatial force field to represent motor output (see below); we also increased the laser power in 2.5 to 15% increments, over 4 to 8 power levels, to examine the effects of power increase on the force field structure (see Supplementary Information, Fig. S2A). We found that a choice of 3% maximum-to-baseline difference in our definition of force threshold above would ensure that the force onset values from the two sets of animals were comparable despite the difference in noise level in the forces measured from the two animal sets.

### Motor outputs represented as spatial force fields

As in earlier studies on motor primitives (e.g. refs 6 and 23), the motor output elicited at each spinal stimulation locus was represented as a time-invariant field of force vectors defined over the workspace of the mouse hind-limb. In order to depict the force pattern resulting from the stimulation, we computed, for each ankle location, the active force profile by subtracting the baseline force from the raw recorded force; this subtraction was done separately for the x- and y-force components. All force fields in subsequent analysis were constructed using the active force profiles. In both mouse lines, our optogenetic stimulation consistently evoked a force response whose magnitude became relatively stable at ~50 ms after stimulation onset (Fig. 1C) (Fig. S3). Thus, to construct a time-invariant field, we obtained the force vector for each ankle location by averaging the force recorded from 50 to 200 ms after stimulation onset.

In our experiments, because of inter-animal variability in hind-limb geometry, different mice were recorded over grids of different sizes (15–36 grid points; mean = 23.3). To facilitate comparison of results across animals and to clarify the spatial structure of the force fields, we resampled all force fields of all animals by linear interpolation so that every field in the data set was composed of vectors defined over an 8 × 8 grid (i.e., a total of 64 vectors). This interpolation, performed separately for the x- and y-components, was accomplished using the Matlab functions ndgrid and griddata (linear option).

### Spinal topography of force fields

To see how different force fields may be topographically represented in the spinal cord, for each mouse type we categorized the force fields obtained from all animals and stimulation loci into clusters, and examined the spatial distribution of the stimulation loci for each cluster along the spinal cord. For this force-field clustering, a force field was included in the analysis only if it was elicited by a single laser beam, obtained with the lowest laser power used in the experiment for the same spinal stimulation locus, and was the first field recorded in the experiment at that stimulation locus with the same stimulation power. These selection criteria ensure that only the force fields generated by the stimulation of the smallest possible volume of neural tissue were used; as a result, the topography obtained is more likely to reflect the spatial organization of functionally distinct spinal networks for the different force fields.

Clustering of the force fields was accomplished using the *k*-means method implemented by Matlab (kmeans; Statistics toolbox). The vectors within each field were first normalized to the maximum vector-magnitude in the field. Then, the x- and y-components of the 64 vectors in each field were reordered into a single array of 64 × 2 = 128 components, so that every force field was represented as a single data point in a 128-dimensional space in which the clustering algorithm was implemented, with distances defined using the squared-Euclidean measure. We repeated the clustering algorithm 500 times, each time with a different set of initial cluster centroids. The repetition with the smallest sum of point-to-cluster-centroid distances was selected for analysis of force-field topography.

The *k*-means algorithm requires the number of clusters to be specified *a priori*. To identify the optimal number of clusters, we first ran the algorithm with the number of clusters ranging from 2 to 30. For every number of clusters, we calculated the silhouette values for all data using Matlab (silhouette; Statistics toolbox). The silhouette value is a measure of how tight a data point is matched to its own cluster relative to the neighbouring clusters; the number of clusters selected is more likely to be valid if most data have high silhouette values^56^. For the Thy1 data set, we chose the best number of clusters by finding the one with the highest median silhouette values. For the Chat data set, we chose the best number of clusters by finding the one with a median silhouette value closest to the maximum Thy1-silhouette value. This matching ensures that clusters in both data sets had the same degrees of within-cluster data spread relative to between-class spread.

When examining the spinal topographic map of the force-field clusters for Thy1, we also quantified the degree of spatial overlap between the ranges of spinal locations for every pair of force-field clusters. For every Thy1 mouse studied, we defined the spinal range for each force-field cluster by noting the most anterior and most posterior stimulation loci that produced force fields belonging to the cluster. Then, we calculated the percentage of this range that overlapped with the range of every other cluster. This percentage was then averaged across all animals.

In the force field data sets we obtained from the two mouse types, we also observed that the Chat fields tended to be composed of parallel force vectors while the Thy1 fields, of non-parallel vectors. We therefore also quantified the degree for a force field to exhibit parallel vectors by first calculating the azimuth angle of every vector in the field, and then calculating the circular standard deviation of this angle across all vectors in the field using the Matlab function circ_std (Circular Statistics toolbox by Philipp Berens, available at Matlab Central File Exchange). For a field with completely parallel vectors, this standard deviation should be 0. Statistical significance of the difference in this quantity between every Chat and Thy1 force fields was assessed using Ansari-Bradley two-sample test for equal dispersions, executed using the Matlab function ansaribradley (Statistics toolbox) after the angles in each field were median-centred. Statistical significance of the between-group difference in standard deviation was subsequently assessed using the U-test (α = 0.05).

### Equilibrium point analysis

In previous studies on spinal motor primitives in the frog (e.g. refs 6, 7, 8), many force fields were characterized by the presence of a single equilibrium point (EP) at which no net active force could be elicited at the ankle by the stimulation. Here, we followed the steps outlined below for automatically detecting the EP’s position. If a force field contained a vector whose magnitude was <2.5% of the maximum vector magnitude in the field, the location of that vector was defined to be an EP. If such a vector did not exist, it remains possible that the vectors along one of the four borders of the workspace were sufficiently convergent so that they defined a “virtual” EP outside of the workspace, but close to the workspace boundary, at the point where the vector lines intersected. Following the steps described in Overduin *et al*.^20^ (their Fig. 1B), we located this virtual EP by first identifying the workspace border whose vectors could possibly define a virtual EP (the “border of interest”, to be described next). Then, we found the intersection point of the lines defined by the directions of every pair of vectors within the set of vectors lying on the border of interest; the virtual EP was then located by averaging the x- and y-coordinates of all intersection points. The border of interest was identified by first finding the vector sum of all vectors in the field; the rostral, caudal, dorsal, or ventral border was selected depending on to which axis the vector sum pointed closest. If the horizontal (or vertical) distance between the virtual EP and the workspace boundary was larger than half of the workspace’s width (or height), then the force field was regarded as possessing no virtual EP. All force fields elicited by a single laser beam were included in this EP analysis. The spatial distributions of the EPs from the Thy1 and Chat animals were compared.

### Model of force-field summation

Distinct motor primitives may be summed together, through linear combination, to produce new patterns of motor output^1^. In this study, we also explored to what extent the optogenetically elicited force fields may be linearly combined to generate new force fields. In both mouse lines, two force fields were first elicited by respective stimulation of two different spinal loci; then, a third force field was elicited by two laser beams concurrently delivering light pulses, at the same frequency and pulse width, to the same two spinal loci. The distance between the two stimulation loci ranged from ~2 to ~7 mm. Stimulation intensities of the two laser beams were also varied to generate a variety of force-field combinations.

The force field resulting from co-stimulation, 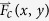, was compared with a linear summation of the two separately elicited force field, 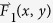 and 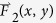. Specifically, we modelled 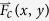 to be generated according to the following equation:


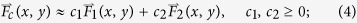


where c_1_ and c_2_ are non-negative scaling coefficients, found by regressing 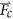 against 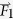 and 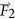. For each force field, the x- and y-components of the 64 vectors were rearranged into an array of 128 components; regression was then implemented in this 128-dimensional space using Matlab (lsqnonneg; Optimization toolbox).

### A similarity measure for force fields

Validity of the model defined by equation (1) was tested by evaluating the similarity between the experimentally derived 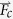 and the computed 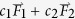. For two vectors at corresponding locations of two different force fields, the dissimilarity between them was measured by the magnitude of the difference vector relative to the sum of the magnitude of the two vectors. We then defined the dissimilarity between the two force fields by the average of this vector-dissimilarity value across all vector pairs, but with this average weighted by the magnitude sum of the two vectors so that this measure would be less sensitive to noise in small-magnitude vectors^7^. Since the field-dissimilarity value ranges from 0 to 1, field-similarity can be conveniently defined by subtracting the dissimilarity value from 1. Thus, accounting for all of the above considerations, for any two force fields 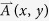 and 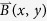, each comprising 64 vectors, the similarity between them (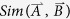) can be calculated by the following formula:


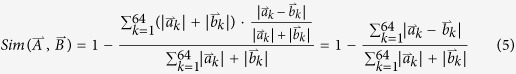


where 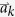 and 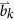 are the *k*th vectors from 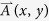 and 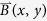, respectively.

## Additional Information

**How to cite this article**: Caggiano, V. *et al*. An Optogenetic Demonstration of Motor Modularity in the Mammalian Spinal Cord. *Sci. Rep*. **6**, 35185; doi: 10.1038/srep35185 (2016).

## Supplementary Material

Supplementary Information
