# Supplementary material for: An Optogenetic Demonstration of Motor Modularity in the Mammalian Spinal Cord
Source: Sci Rep. 2016 Oct 13;6:35185. doi: 10.1038/srep35185 (PMC5062376; doi:10.1038/srep35185)
Supplement: Supplementary Information [file srep35185-s1.pdf]

SUPPLEMENTARY INFORMATION

AN OPTOGENETIC DEMONSTRATION OF MOTOR MODULARITY IN THE MAMMALIAN SPINAL CORD

Vittorio Caggiano*, Vincent C. K. Cheung*, Emilio Bizzi

* = equal contributions

The analysis in the main text of motoneuronal and interneuronal spinal topography considered only the force fields elicited by the lowest laser power used at each spinal locus examined. We also investigated how sensitive the force-field structure was to the power of the laser used in both mouse types. At various spinal loci, we successively increased the laser power at 2.5 to 15% increments, over 4 to 8 power levels. We then summarized the change of force-field structure induced by power increase by examining the change in the x- and y-components of the force-field vector sum, with its magnitude normalized to unity (with increasing x corresponding to the anterior direction, and increasing y to the dorsal), and also, the change in the average vector magnitude across all vectors in the field, as a function of power level. In both Chat and Thy1 mice, increasing stimulation power had the effect of increasing the average magnitude of the force-field vectors (Fig. S1A & S1B, right panel). In the Chat mouse, the overall flow direction of the force-field vectors was not appreciably changed by power increase, as suggested by the relatively constant x- and y-components of the force-field vector sum across the power levels (Fig. S1A, left and middle panels). In the Thy1 mouse, however, increasing laser power decreased the x-component (Fig. S1B, left panel), and either increased or decreased the y-component of the vector sum (Fig. S1B, middle panel). Thus, for the Thy1 mouse, increasing the stimulation power tended to shift the flow of the force-field vectors toward the posterior direction (i.e., decreasing x) by vector rotation through the dorsal (i.e., increasing followed by decreasing y) or ventral direction (i.e., decreasing followed by increasing y).

We show in Fig. S1 two examples of how increasing the stimulation power can alter the Thy1 force field. In the first example, as power was increased the force vectors rotated from the anterior to the dorsal directions (Fig. S1C). In the second example, increasing power had the effect of rotating the vectors from the anterior, then to the ventral, and finally to the posterior/dorsal directions (Fig. S1D).

In addition, we also analysed how increasing the stimulation power at the same spinal loci may alter the onset time of force emergence and the degree of vector divergence within the force field, the latter quantified by the circular standard deviation (SD) of the force-vector azimuth angle. In Chat, as compared with Thy1 a greater minimum power laser (usually at 40-50 mW with respect to ~10 mW necessary in the Thy1) was needed to evoke observable hindlimb force; but over the range of power tested, the force onset time remained constant at 6.6 ± 2.0 ms (mean ± SD; Fig. S2A, red; see also Fig. 1D). In Thy1, as laser power was increased, force onset time decreased from 11.4 ± 5.4 ms (Fig. S2A, blue, bin 1; Fig. 1D) to 4.9 ± 1.2 ms (bin 3) (multiple comparison after Kruskal-Wallis, $p < 0.01$). In fact, the Thy1-onset time was significantly greater than the Chat onset times only at the lowest power ($p < 0.01$), but not at other powers ($p > 0.05$). The higher Thy1-onset time recorded at the lowest power argues that at this stimulation intensity, the force evoked likely resulted from activations of interneurons, which could be one to several synapses away from the motoneurons. At higher power

intensities, the motoneurons were recruited in addition to the interneurons, thus resulting in the similar onset times between Chat and Thy1.

On the other hand, in both mouse strains the circular SD of the vector azimuth angles did not vary over the range of stimulation power tested ($p > 0.05$; Fig. S2B). The Thy1 values, however, were overall greater than the Chat values ($p < 0.01$; Fig. S2B, *). This result illustrates that even when the motoneurons were activated along with the interneurons, the resulting force fields were still more complex in structure than the Chat fields, further suggesting that the divergence of vectors in Thy1 owes its origin to interneuronal activations.

Force fields emerged over time and assumed a more stable configuration only after 50 ms from light onset. In Fig. S3, we depict typical time courses of how force fields evolved over the 200-ms stimulation time, for a Chat field (Fig. S3A), a convergent Thy1 field (Fig. S3B), and a divergent Thy1 field (Fig. S3C). The force field's structure stabilized by 25-50 ms for Chat, and by 50-75 ms for the Thy1 fields. Prompted by these observations, in all of our analysis for both mouse strains, we constructed a time-invariant force field by obtaining the force vector for each ankle location, and then averaging the force recorded from 50 to 200 ms after stimulation onset (see Methods of main text).

SUPPLEMENTARY FIGURE

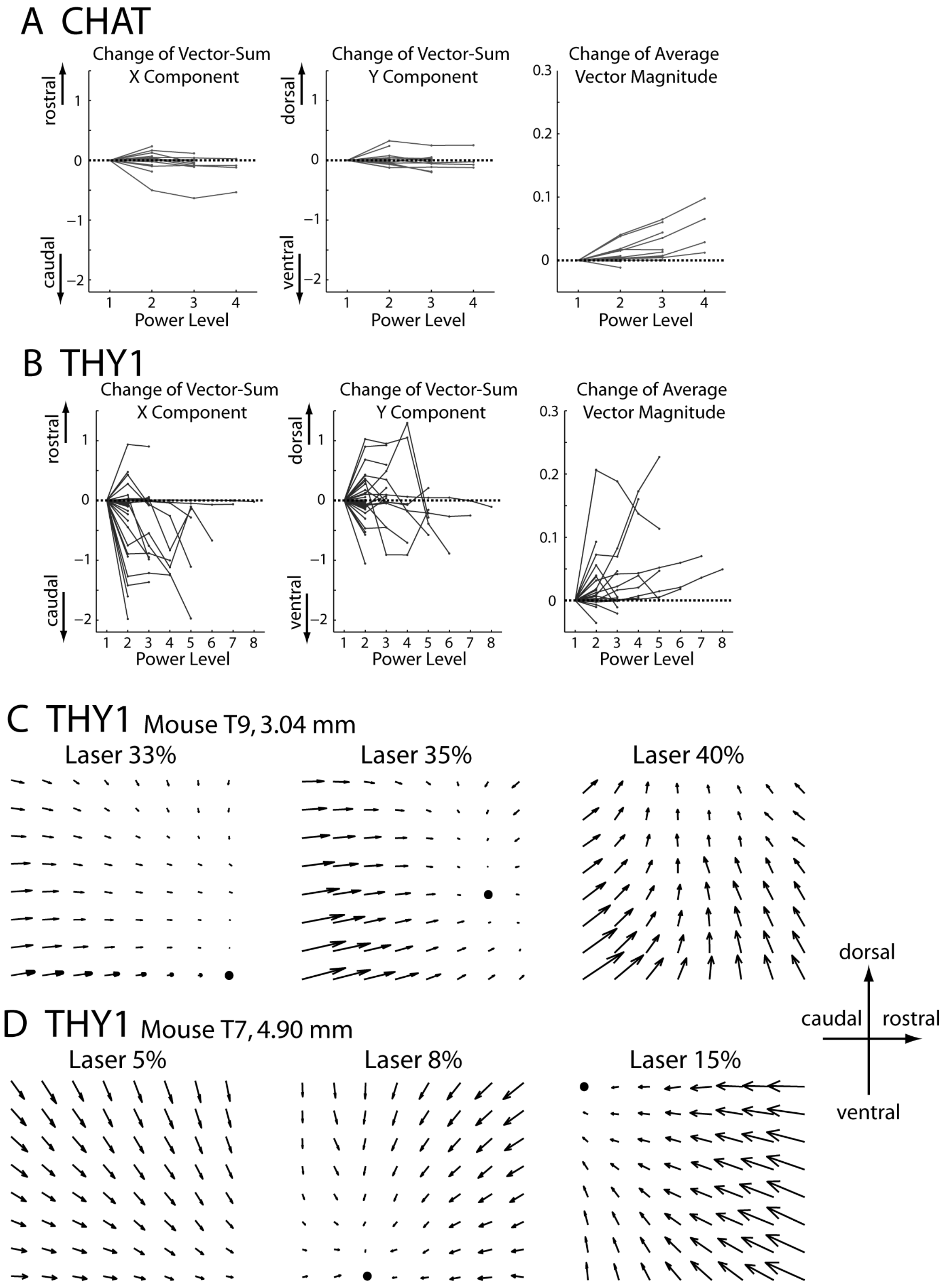

FIGURE S1. Effect of power increase on the force fields in both Chat and Thy1 mice. ***A,*** In Chat, increasing the stimulation power did not alter the orientation of the force field, as evidenced by the lack of change of both the x- (left panel) and y- (middle panel) components of the vector sum as power was increased. Increasing the power, however, did increase the magnitude of the force vectors (right panel). ***B,*** In Thy1, increasing the power tended to shift the

force field to a more caudal orientation, as evidenced by a decrease in the x-component (left panel), but a variable change in the y-component (middle panel), of the field vector sum as power was increased. ***C, D,*** Two examples, from two different Thy1 mice and two stimulation loci, showing how increasing the laser power shifted the force field to a more caudal orientation, through a rotation towards either the dorsal (panel *C*) or ventral (panel *D*) directions.

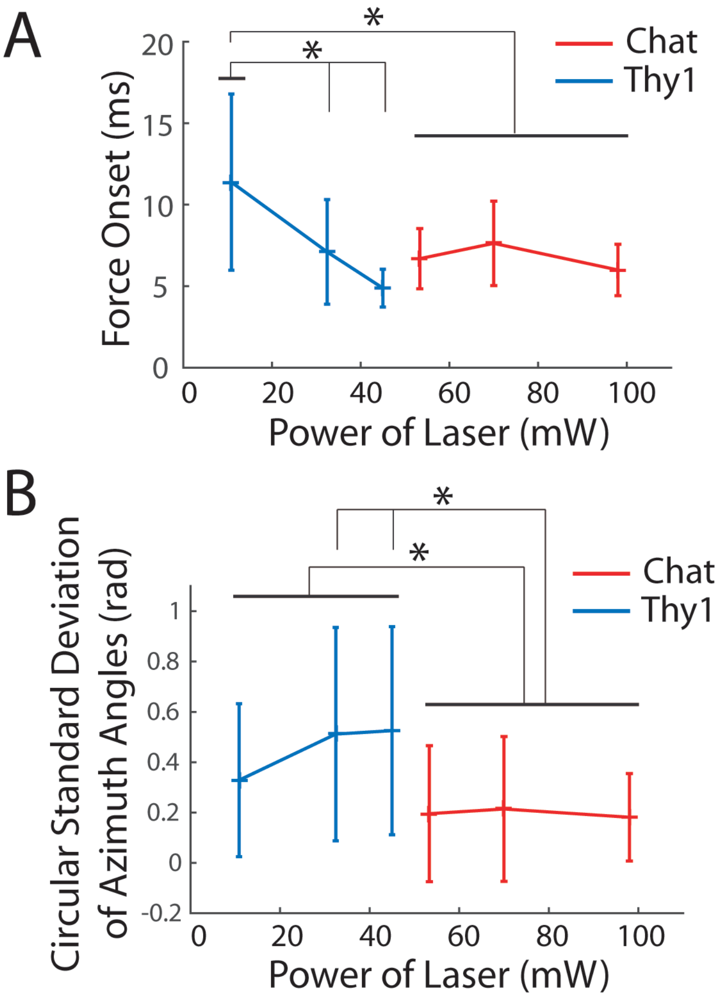

FIGURE S2. Effect of power increase on the force onset time and within-field vector divergence in Thy1 and Chat. ***A,*** In Thy1 (blue), increasing stimulation power decreased the force onset time (*; multiple comparison after Kruskal-Wallis, $p < 0.01$) while in Chat (red), onset time remained fairly constant over the range of power tested. Only at the lowest power was the Thy1-onset time significantly different from the Chat onset time (*; $p < 0.01$). This result suggests that at minimum power, the force field evoked in Thy1 likely results from only interneuronal activations. Note that as compared with Thy1, Chat required larger power intensities for producing observable forces. ***B,*** In both Thy1 and Chat, the circular SD of the vector azimuth angle did not vary over the range of power tested ($p > 0.05$). But the Thy1 values were significantly greater than the Chat values (*; $p < 0.01$). This suggests that interneuronal activations were sufficient to produce force fields more complex in structure than the Chat fields.

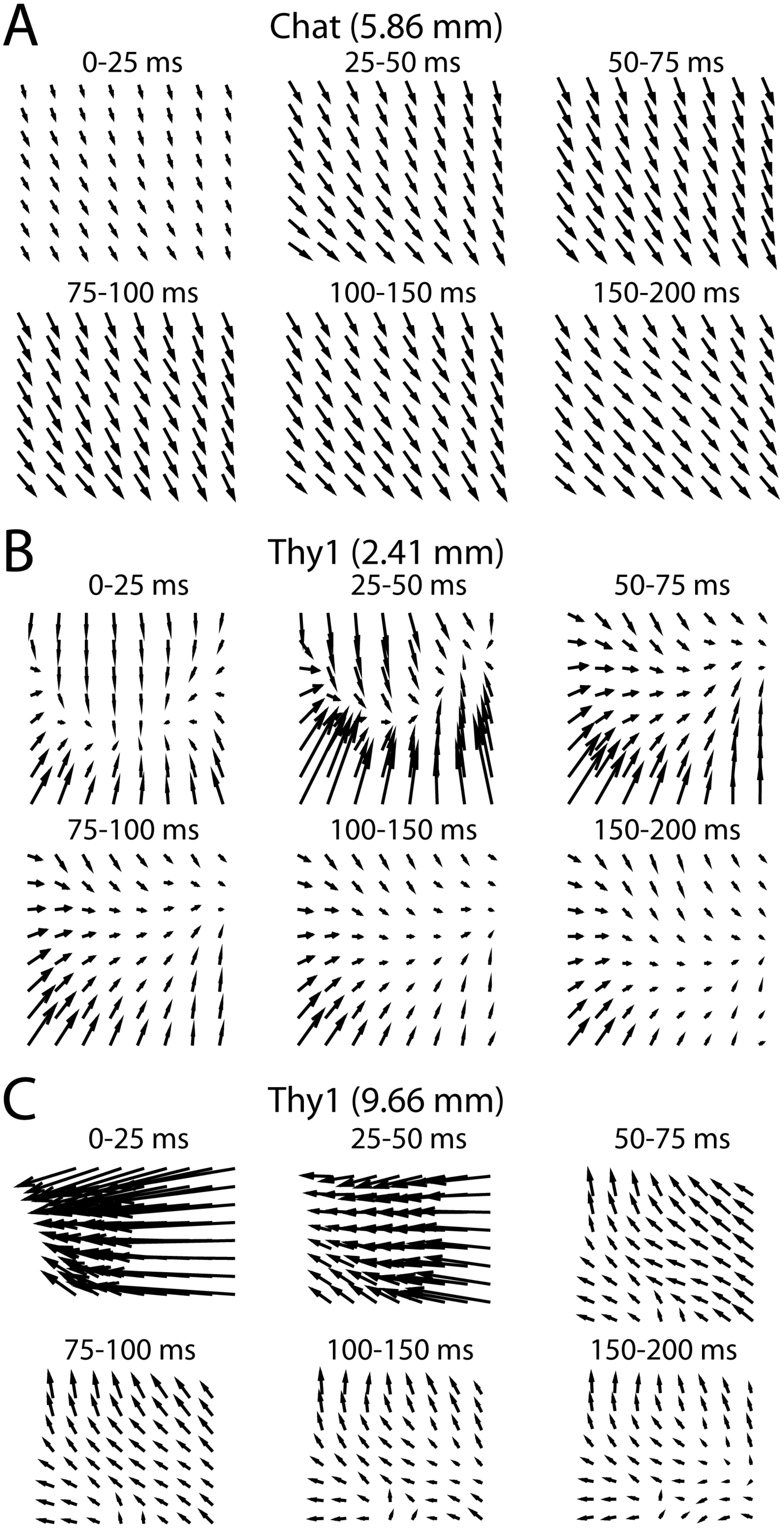
A
Chat (5.86 mm)
0-25 ms
25-50 ms
50-75 ms
75-100 ms
100-150 ms
150-200 ms
B
Thy1 (2.41 mm)
0-25 ms
25-50 ms
50-75 ms
75-100 ms
100-150 ms
150-200 ms
C
Thy1 (9.66 mm)
0-25 ms
25-50 ms
50-75 ms
75-100 ms
100-150 ms
150-200 ms

Figure S3. Time courses of how force fields evolved after laser stimulation onset. We show the time courses of ***A***, a Chat force field (recorded at spinal stimulation locus of 5.86 mm), ***B***, a convergent Thy1 force field (at 2.41 mm), and ***C***, a divergent Thy1 force field (at 9.66 mm). The six fields in each time course were constructed by averaging force vectors (recorded at 20,000 Hz) over the time ranges of 0-25, 25-50, 50-75, 75-100, 100-150, and 150-200 ms, respectively. In all three examples, the structure of the force field stabilized by 50-75 ms.
